# Impact of regionalizing ST‐elevation myocardial infarction care on sex differences in reperfusion times and clinical outcomes

**DOI:** 10.1002/clc.23658

**Published:** 2021-06-08

**Authors:** Erin Rayner‐Hartley, Graham C. Wong, Cassandra Fayowski, John A. Cairns, Joel Singer, Terry Lee, Tara Sedlak, Karin H. Humphries, Michele Perry‐Arnesen, Martha Mackay, Christopher B. Fordyce

**Affiliations:** ^1^ Division of Cardiology University of British Columbia Vancouver British Columbia Canada; ^2^ Royal Columbian Hospital, Division of Cardiology Fraser Health Authority Surrey British Columbia Canada; ^3^ Vancouver Coastal Health Authority Vancouver British Columbia Canada; ^4^ Division of General Internal Medicine Western University London Ontario Canada; ^5^ School of Population and Public Health University of British Columbia Vancouver British Columbia Canada; ^6^ Centre for Health Evaluation and Outcome Sciences (CHEOS), Providence Health Care Research Institute University of British Columbia Vancouver British Columbia Canada; ^7^ School of Nursing University of British Columbia Vancouver British Columbia Canada

**Keywords:** sex differences, ST‐elevation myocardial infarction, systems implementation

## Abstract

**Background:**

Women with ST‐elevation myocardial infarction (STEMI) treated with primary percutaneous coronary intervention historically experience worse in‐hospital outcomes compared to men.

**Hypothesis:**

Implementation of a regional STEMI system will reduce care gaps in reperfusion times and in‐hospital outcomes between women and men.

**Methods:**

1928 patients (413 women, 21.4%) presented with an acute STEMI between June 2007 and March 2016. The population was divided into an early cohort (n = 728 patients, 2007‐May 2011), and a late cohort (n = 1200 patients, June 2011–2016). The primary endpoints evaluated were reperfusion times and in‐hospital outcomes.

**Results:**

Compared to men, women experienced significant delays in first medical contact (FMC) to arrival at the emergency room (26.0 vs. 22.0 min, p < 0.001) and FMC‐to‐device (109 vs. 101 min p = 0.001). Women had higher incidences of post‐PCI heart failure and death compared to men (p < 0.05). Following multivariable adjustment, no mortality difference was observed for women versus men (adjusted OR; 0.82; 95% confidence interval [CI], 0.51–1.34; p = 0.433) or for early versus late cohorts (adjusted OR; 1.04; 95% CI, 0.68–1.60; p = 0.856).

**Conclusion:**

Following STEMI regionalization, women continued to experience significantly longer reperfusion times, although there was no difference in adjusted mortality. These results highlight the ongoing disparity of STEMI care between women and men, and suggest that regionalization alone is insufficient to close sex‐based care gaps.

## 1 INTRODUCTION

Cardiovascular disease is a leading cause of mortality in both women and men in Canada. Women presenting with acute myocardial infarction (AMI) are older, have more co‐morbidities (greater prevalence of diabetes mellitus, hypertension and heart failure [HF]) and higher mortality.^1^, ^2^, ^3^, ^4^, ^5^, ^6^, ^7^, ^8^, ^9^, ^10^, ^11^, ^12^, ^13^, ^14^, ^15^, ^16^ However, it is unclear whether female sex is an independent predictor of mortality in AMI after adjusting for differences in age and other comorbidities.^1^, ^11^, ^14^

Primary percutaneous coronary intervention (pPCI) is the recommended reperfusion therapy for acute ST‐elevation myocardial infarction (STEMI) in PCI‐capable hospitals, and also for patients with high‐risk features or contraindications to fibrinolysis in PCI non‐capable hospitals.^17^ For a regional STEMI system in an urban setting, the target time intervals for first medical contact (FMC) to device are less than 90 min for those presenting to a PCI‐capable hospital and less than 120 min for those presenting to a PCI‐non‐capable hospital.^18^ Women are less likely to achieve these target time intervals compared to men.^2^, ^19^

Little data exists regarding the impact of regionalization of STEMI care on addressing care gaps in the management of women, including rates of timely reperfusion. The Vancouver Coastal Health Authority (VCHA) Regional STEMI Committee implemented a standardized STEMI protocol in two phases between 2007–2011 that increased regional access to pPCI and decreased overall reperfusion times.^20^ We undertook to describe differences in clinical characteristics, temporal trends in reperfusion times and in‐hospital outcomes between women and men with STEMI receiving pPCI, before and after STEMI regionalization. We hypothesized that regionalization of STEMI care would reduce care gaps, including reperfusion times, and improve outcomes.

## 2 METHODS

This study was a retrospective analysis of data collected between June 2007 and March 2016 on all consecutive patients with STEMI who presented to a VCHA hospital and received PCI (both the initial intended and actual reperfusion strategy was PCI). All baseline characteristics, presenting and in‐hospital clinical data were manually extracted from patient charts, as previously described.^20^ Patients with long ischemic times (>12 h from symptom onset) were excluded. Patients were grouped into two cohorts, according to the phase of implementation of the pPCI system. The early cohort (June 2007 to May 2011) was assembled during the initial phase of implementation of pPCI in VCHA, which included standardization of STEMI reperfusion protocols at VCHA hospitals, prehospital ECG detection of STEMI with redirection to PCI‐capable hospitals and development of a 1‐step “Hot STEMI” activation process to minimize the time required to activate the cardiac catheterization laboratory team. The late cohort (June 2011–May 2016) was assembled following the adoption of region‐wide pPCI as the reperfusion modality of choice, refinement of the interfacility transfer protocols and further adjustment of the “Hot STEMI” activation process, as previously described in detail.^20^ Sex‐based comparisons between the outcomes of the two cohorts were undertaken to assess the impacts of the sequential development and implementation of our regionalized STEMI program on potential care gaps between men and women. This study was approved by the University of British Columbia Clinical Research Ethics Board (No. H17‐01580). As the study used only existing de‐identified patient records and was collected as part of a quality improvement initiative, individual patient informed consent was not required.

### 2.1 Outcomes

The primary outcomes were reperfusion times and in‐hospital outcomes (cardiogenic shock, HF and death). Comparisons between the sexes were then made for (a) the entire cohort, (b) within the early cohort, and (c) within the late cohort and for all patients between the early and late cohorts.

### 2.2 Definitions

The definitions of all outcomes and reperfusion intervals have been described previously.^20^ In brief, HF on presentation was defined as physician documentation of HF or any description of rales or pulmonary edema on physical exam or chest x‐ray, jugular venous distention, unusual dyspnea on light exertion, Killip class 2, 3, or 4 or presence of an S3. Cardiogenic shock on presentation was defined as a sustained (> 30 min) episode of systolic blood pressure < 90 mm Hg, and/or cardiac index <2.2 L/min/m^2^ determined to be secondary to cardiac dysfunction, and/or the requirement for parenteral inotropic or vasopressor agents or mechanical support (e.g., intra‐aortic balloon pump, extracorporeal circulation, ventricular assist devices) to maintain blood pressure and cardiac index above those specified levels. We used the same definitions for in‐hospital (i.e., following admission) HF and cardiogenic shock. Major bleeding was defined as documentation in the medical record of a suspected bleeding event during the index hospitalization that was associated with significant blood loss (hematocrit drop of at least 10% and/or hemoglobin drop of at least 3 g/dl), transfusion of whole blood or packed red blood cells, or use of a surgical or procedural intervention to stop bleeding.

### 2.3 Reperfusion time intervals

FMC was defined as the time at which a health care provider was at the patient's side. For patients arriving via ambulance, this was defined as the time at which a paramedic first assessed the patient. For patients who transported themselves to the hospital, FMC was defined as the time at which they were registered in the emergency department.

### 2.4 Statistical analysis

Baseline demographics and presenting clinical characteristics were summarized using means (± *SD*), medians (with interquartile range), or proportions as appropriate. Between‐group comparisons were made using the Wilcoxon rank sum test or *t* test for continuous variables, and the χ^2^ or Fisher's exact test for categorical variables. To account for the possibility of changes in baseline clinical characteristics during the study period, a multivariable logistic regression analysis for in‐hospital mortality was constructed. The following prognostically important baseline variables were chosen for the model: age, diabetes, HF on presentation and vital signs on presentation (heart rate and systolic blood pressure). For all variables / outcomes, there was a < 2% missingness, with the exception of cardiogenic shock. Adjusted odds ratios (ORs) with their 95% confidence intervals (CI) were calculated. For each comparison, a two‐sided p value of <0.05 was considered statistically significant. All statistical analyses were performed using SAS, version 9.4 (SAS Institute, Cary, NC).

## 3 RESULTS

### 3.1 Baseline characteristics

**FIGURE 1:**
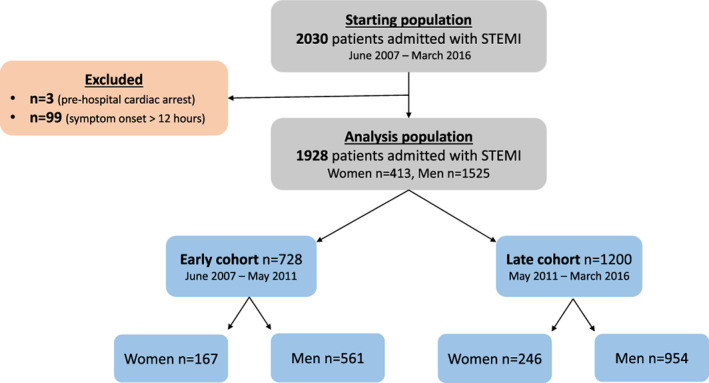
Patient flow diagram. PCI, percutaneous coronary intervention, STEMI, ST‐elevation myocardial infarction

Between May 2007 and March 2016, 2030 patients presented to VCHA hospitals (both PCI capable and non‐capable) with the diagnosis of acute STEMI and were treated with pPCI. 102 patients were excluded ‐ 99 due to symptom onset >12 h and three due to unknown symptom onset time. In 1928 patients (413 [21.4%] women) were included in the final analysis; of these there were 728 (167 [22.9%] women) in the early cohort; and 1200 (246 [20.5%] women) in the late cohort (Figure 1). At baseline, compared to men, women were older (mean age 73.1 vs. 62.7, p < 0.001), and had higher rates of preexisting hypertension, previous cerebrovascular accident (CVA) and history of HF. Overall, women presented in HF more frequently (8.8% vs. 5.8%, p = 0.029) than their male counterparts. (Table 1). The majority of these differences between sexes were seen in both the early and late cohorts.

**TABLE 1:** Baseline characteristics

Variable	All (*n* = 1928)	Women (*n* = 413)	Men (*n* = 1515)	p
Demographics				
Mean age, years (*SD*)	65.0 (13.1)	73.1 (12.8)	62.7 (12.3)	<0.001
Medical history				
Current/recent smoker, *n* (%)	544 (28.5)	84 (20.5)	460 (30.6)	<0.001
Hypertension, *n* (%)	1016 (51.3)	267 (65.1)	749 (49.8)	<0.001
Dyslipidemia, *n* (%)	813 (42.5)	170 (41.5)	643 (42.8)	0.625
Diabetes, *n* (%)	377 (19.7)	92 (22.4)	285 (19.0)	0.120
Prior MI, *n* (%)	274 (14.3)	49 (12.0)	225 (15.0)	0.124
Prior heart failure, *n* (%)	51 (2.7)	17 (4.2)	34 (2.3)	0.035
Prior PCI, *n* (%)	204 (10.7)	30 (7.3)	174 (11.6)	0.014
Prior CABG, *n* (%)	44 (2.3)	5 (1.2)	39 (2.6)	0.100
Prior TIA / stroke, *n* (%)	129 (6.8)	52 (12.7)	77 (5.1)	<0.001
Prior PVD, *n* (%)	56 (2.9)	17 (4.2)	39 (2.6)	0.098
Clinical presentation				
Heart failure, *n* (%)	123 (6.5)	36 (8.8)	87 (5.8)	0.029
Cardiogenic shock, *n* (%)^a^	151 (10.6)	33 (11.3)	118 (10.5)	0.692
Initial median creatinine, mmol/L (IQR)	93 (79–113)	82 (69–101)	97 (82–115)	<0.001
Initial median hemoglobin, g/L (IQR)	142 (131, 153)	128 (119, 138)	146 (137, 155)	<0.001

Abbreviations: CABG, coronary artery bypass graft; CVA, cerebral vascular accident; MI, myocardial infarction; PCI, percutaneous coronary intervention; PVD, peripheral vascular disease; *SD*, standard deviation; TIA, transient ischemic attack.

^a^ Data for this outcome was missing in 507 patients.

### 3.2 Reperfusion time intervals

**FIGURE 2:**
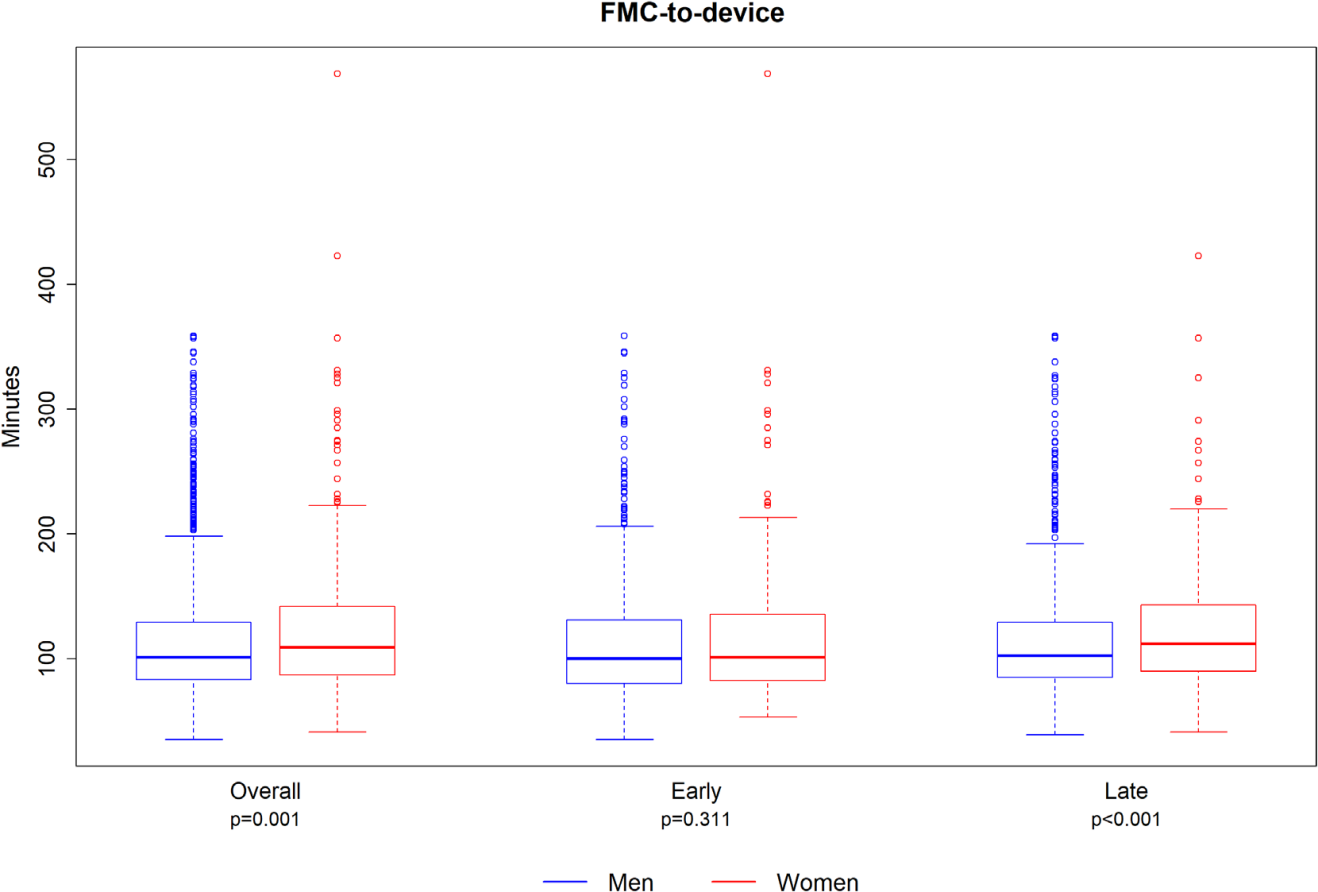
Reperfusion time intervals. FMC, first medical contact; ED, emergency department

Median time from symptom onset to first medical contact (FMC) time was longer for women (62.0 min vs. 50.0 min, p = .002) over all, and in both the early (65.0 min vs. 51.0 min, p = 0.022) and the late cohort (60.0 min vs. 49.0 min, p = 0.031) (Supplementary Figure S1). FMC to emergency department (ED) arrival was longer for women overall (26.0 min vs. 22.0 min, p = 0.001) in both the early and late cohorts. After ED arrival, there were no further significant differences between women and men for the sub‐intervals of ED arrival to on‐cath‐lab‐table time (overall cohort p = 0.241), on‐table to arterial puncture time (overall cohort p = 0.228) or puncture to device time (overall cohort p = 0.494). FMC‐to‐device time was significantly longer for women overall (109 min vs. 101 min, p < 0.001), accounted for by significantly longer delays in the late cohort only (112 min vs. 102 min, p < 0.001) (Figure 2). These findings were similar regardless of whether patients presented to PCI‐capable or non‐capable hospitals. Overall, FMC‐to‐device was achieved within target times (≤ 90 min at PCI‐capable centers; ≤ 120 min at PCI non‐capable centers) in 37.0% of women and 44.7% of men (p = 0.005). Comparison of target times in the early cohort showed no difference between the sexes (women 39.5% vs. men 42.5%, p = 0.5) but in the late cohort there was a significant difference (women 35.9% vs. men 46%, p = 0.003).

### 3.3 Clinical outcomes

#### 3.3.1 Univariate analysis

**FIGURE 3:**
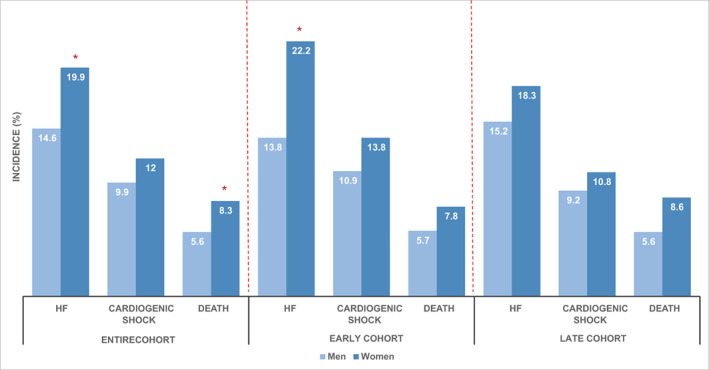
In‐hospital outcomes (heart failure [HF], cardiogenic shock, death) in the overall, early and late cohorts. *denotes statistically significant differences between women and men

Development of HF was significantly more common in women than men in the overall (19.9% vs.14.6%, p = 0.011) and early (22.2% vs. 13.8%, p = 0.009) cohorts, but not in the late cohort (18.3% vs. 15.2%, p = 0.244; Figure 3). There was no significant difference in the incidence of cardiogenic shock between sexes. However, women had a higher incidence of in‐hospital death overall compared to men, (8.3% vs. 5.6%, p = 0.046), although this did not reach statistical significance in either the early or late cohorts. (Figure 3). The findings were unchanged in a sensitivity analysis which considered the composite outcome of death or CHF, as well as death or cardiogenic shock (Supplementary Table S1).

#### 3.3.2 Multivariable‐adjusted analysis

Following adjustment for age, diabetes, HF on presentation and vital signs on presentation (heart rate and systolic blood pressure), we observed no significant difference in in‐hospital mortality in women compared to men in the overall cohort (adjusted OR; 0.82; 95% confidence interval [CI], 0.51–1.34; p = 0.433) or in the early compared to late cohorts (adjusted OR; 1.04; 95% CI, 0.68–1.60; p = 0.856).

## 4 DISCUSSION

We explored sex differences in clinical characteristics and temporal trends in reperfusion times and in‐hospital outcomes in STEMI patients treated with pPCI in our contemporary regional system of care within a provincially‐funded healthcare system. We found that compared to men, women presented with more comorbidities, had longer reperfusion times in the late phases of implementation of our STEMI care system and generally experienced worse unadjusted in‐hospital outcomes. However, these differences did not translate into a mortality difference between sexes once adjusted for clinically important parameters. Our results confirm and extend our current understanding regarding the disparity of STEMI care between women and men, and present opportunities for future improvement.

It remains unclear whether implementation of STEMI systems of care improves management and outcomes in women. Establishment of regional systems has been shown to improve reperfusion times in both sexes and is a Class I recommendation in North American STEMI management guidelines.^18^, ^20^ However, in the current era of pPCI, sex‐based disparities continue to exist in STEMI.^1^ In a review of 2.5 million patients in the National Registry of Myocardial Infarction database, women were significantly less likely to receive reperfusion therapy.^21^ Furthermore, a recent analysis in the United States found that implementing STEMI systems of care did not reduce disparities in reperfusion times between males and females.^15^

Female sex has been reported as an independent predictor of treatment delay in STEMI.^22^ In the current study, we found that women had longer FMC‐to‐device times in the overall and late cohorts as compared to men. In contrast, men had no significant change in FMC‐to‐device times following implementation of this regional system of care. In fact, to our surprise, gaps in FMC‐to‐device times between sexes widened in the late cohort. Our results extend the findings of the STEMI Accelerator project, which evaluated the outcomes of 23 809 patients across 16 regions in the United States and found no difference in the proportion of women treated to guideline goals during the 20‐month study period.^15^ We provide a contemporary, Canadian update to these previously reported results, over a significantly longer follow‐up period.

There may be several reasons that explain the longer reperfusion times and worse outcomes in women, such as less recognizable symptoms on presentation, less pronounced ST‐segment elevation and longer delays after presentation to hospital.^1^, ^23^, ^24^ In our cohort, despite a higher incidence of unadjusted in‐hospital adverse outcomes among women, in‐hospital mortality among women was no longer greater than that in men, after adjustment for important clinical variables. There are several possible explanations for this finding. Advanced age, baseline co‐morbidities and HF at presentation may, in fact, be more important determinants of reperfusion times and mortality, and has been reported recently by our group.^25^ Adjustment for important cardiovascular co‐morbidities has previously been shown to eliminate the difference in mortality between sexes.^1^, ^11^, ^13^, ^14^ Taken together, it appears that women with STEMI are at higher risk than men, not because of their sex, but because of their higher prevalence of several co‐morbidities. Therefore, healthcare providers and administrators should consider these sex differences and target strategies to reduce disparities. For example, Norris et al. recently reported their experience implementing a structured process for considering sex in a clinical practice guideline for the management of STEMI.^26^ Of 175 studies included, only one study met their inclusion criteria to test the applicability of the STEMI guidelines recommendations to both sexes. Their findings suggest that it is both feasible and informative to review the applicability of guidelines to both sexes. Our study further re‐enforces the need to understand and narrow care gaps for female STEMI patients. Implementation of sex‐specific bundles of care for STEMI patients has not been studied; however, this may provide an avenue to further evaluate sex specific treatment approaches.

To our knowledge, this is the most contemporary study describing the impact of a regional STEMI system on the care gap in reperfusion times between sexes. Although regional access to pPCI increased, we unexpectedly noted longer reperfusion times among women with no associated differences in mortality between sexes. This analysis also reinforces the need for ongoing research and education required to eliminate barriers to care for women, including considering a structured framework for sex‐specific evidence and guideline generation, as well as implementation.^25^

## 5 LIMITATIONS

This study has some limitations. First, due to the observational nature of our data, the relationship between implementation of the regional STEMI program and reperfusion times and outcomes may be related to concomitant temporal changes in care processes, such as newer medical therapies. However, we compared groups of patients during the same time period, and therefore these unmeasured temporal factors likely affected both sexes to a similar extent. Since STEMI regionalization did not specifically target care gaps between sexes, the potential impact of a regional program to improve outcomes between sexes may be underestimated. Second, our results are applicable mainly to STEMI systems similar to ours, namely, those with multiple community hospitals in proximity to a pPCI center versus jurisdictions with more widespread access to pPCI. However, in Canada, most catheterization laboratories capable of pPCI are geographically centered around urban areas similar to VCHA. Internationally, the external validity of our findings may vary significantly based on geographic factors. Third, we focused on in‐hospital outcomes and did not capture long‐term outcome data, which may be of interest in further studies.

## 6 CONCLUSIONS

During the phased implementation of our contemporary STEMI system in a large, geographically complex region of British Columbia, Canada, we found that women presenting with STEMI experienced significantly longer reperfusion times compared to men. We unexpectedly noted longer reperfusion times among women in the later phases of implementation of our STEMI reperfusion system. After adjustment for clinically important parameters, no mortality difference was observed between sexes. This suggests that sex alone is not an independent predictor of mortality but rather that women STEMI patients have a higher prevalence than men of several co‐morbidities. These results highlight the ongoing disparity in STEMI care between women and men, and that regionalization alone may be insufficient to bridge care gaps. Implementing sex‐specific strategies to evaluate and mitigate these gaps is needed.

## CONFLICT OF INTEREST

Dr. Fordyce reports grants and personal fees from Bayer, personal fees from Novo Nordisk, personal fees from Boehringer Ingelheim, personal fees from Sanofi, personal fees from Pfizer, personal fees from Amgen, personal fees from Novartis, outside the submitted work. Dr Cairns reports research grants from Edwards Lifesciences and consultant fees from Abbott. All other authors have nothing to disclose.

## Supporting information

**Figure S1** Reperfusion time intervals from symptom onset to device. FMC, first medical contact; ED, emergency department.
**Table S1**. Association between sex and phase with the composite outcomes of CHF or death and cardiogenic shock or death.

## Data Availability

The data underlying this article will be shared on reasonable request to the corresponding author.
