# Supplementary material for: Impact of regionalizing ST‐elevation myocardial infarction care on sex differences in reperfusion times and clinical outcomes
Source: Clin Cardiol. 2021 Jun 8;44(8):1113–9. doi: 10.1002/clc.23658 (PMC8364721; doi:10.1002/clc.23658)
Supplement: Supplementary file 1 — Figure S1 Reperfusion time intervals from symptom onset to device. FMC, first medical contact; ED, emergency department. Table S1. Association between sex and phase with the composite outcomes of CHF or death and cardiogenic shock or death. [file CLC-44-1113-s001.docx]

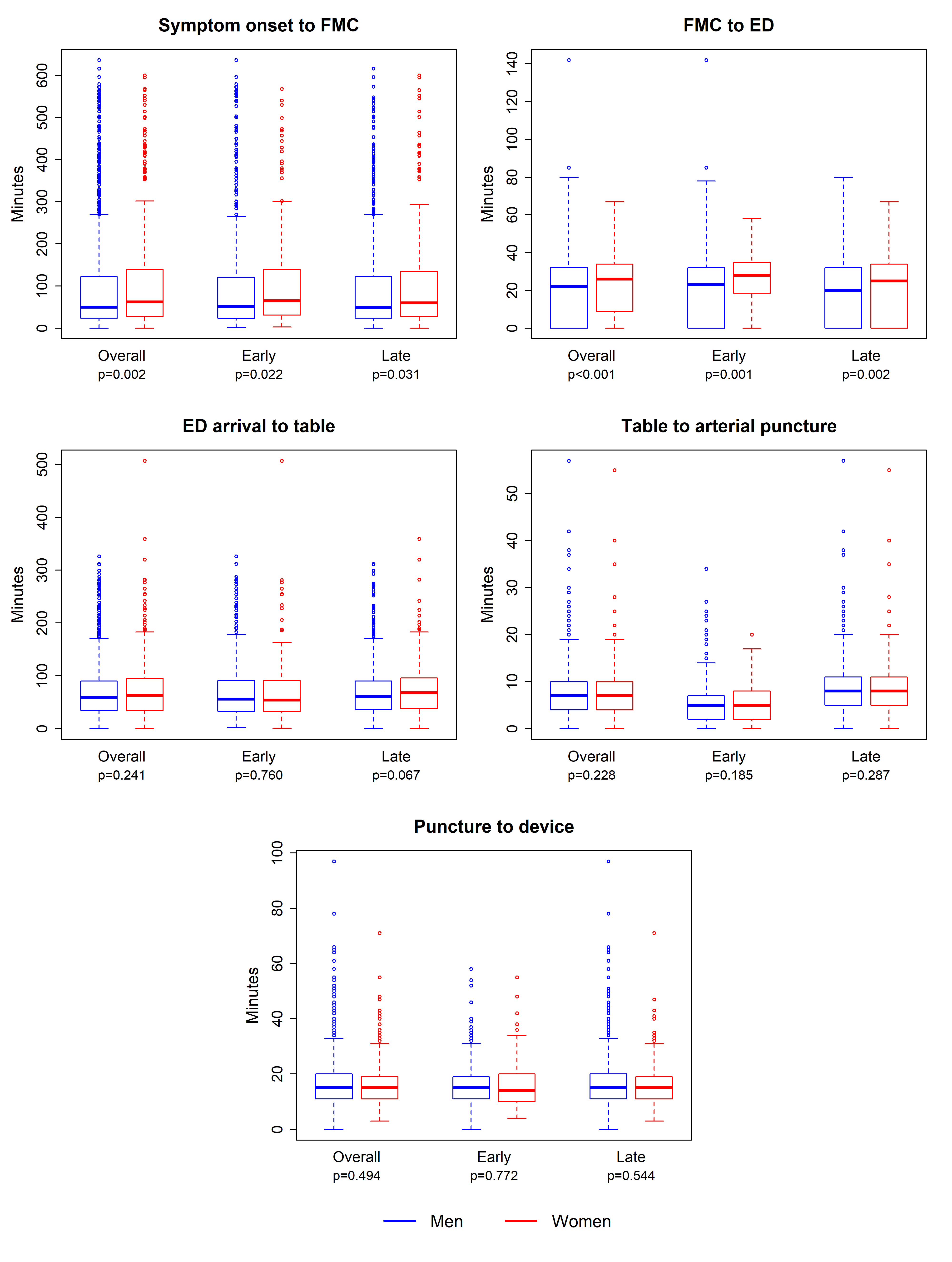


Figure S1. Reperfusion time intervals from symptom onset to device. FMC, first medical contact; ED, emergency department.

Table S1. Association between sex and phase with the composite outcomes of CHF or death and cardiogenic shock or death.

|  | **Overall** | |  | **Early** | |  | **Late** | |  |
| --- | --- | --- | --- | --- | --- | --- | --- | --- | --- |
|  | **Male** | **Female** | **P** | **Male** | **Female** | **P** | **Male** | **Female** | **P** |
| CHF or death, n (%) | 243 (16.2) | 88 (21.6) | 0.012 | 95 (17.0) | 43 (25.7) | 0.011 | 148 (15.8) | 45 (18.7) | 0.282 |
| Cardiogenic Shock or death, n (%) | 159 (10.6) | 54 (13.2) | 0.141 | 68 (12.2) | 27 (16.2) | 0.181 | 91 (9.7) | 27 (11.2) | 0.495 |
